# The role of the obestatin/GPR39 system in human gastric adenocarcinomas

**DOI:** 10.18632/oncotarget.6718

**Published:** 2015-12-22

**Authors:** Begoña O. Alén, Saúl Leal-López, María Otero Alén, Patricia Viaño, Victoria García-Castro, Carlos S. Mosteiro, Andrés Beiras, Felipe F. Casanueva, Rosalía Gallego, Tomás García-Caballero, Jesús P. Camiña, Yolanda Pazos

**Affiliations:** ^1^ Área de Endocrinología Molecular y Celular, Instituto de Investigación Sanitaria de Santiago (IDIS), Complejo Hospitalario Universitario de Santiago (CHUS), Servicio Gallego de Salud (SERGAS), Santiago de Compostela, Spain; ^2^ CIBER Fisiopatología de la Obesidad y Nutrición, Santiago de Compostela, Spain; ^3^ IDIS, CHUS, Santiago de Compostela, Spain; ^4^ Servicio de Anatomía Patológica, CHUS, SERGAS, Santiago de Compostela, Spain; ^5^ Departamento de Ciencias Morfológicas, Universidad de Santiago de Compostela (USC), Santiago de Compostela, Spain; ^6^ Departamento de Medicina, USC, Santiago de Compostela, Spain

**Keywords:** GPR39, obestatin, stomach, adenocarcinoma

## Abstract

Obestatin, a 23-amino acid peptide encoded by the ghrelin gene, and the GPR39 receptor were reported to be involved in the control of mitogenesis of gastric cancer cell lines; however, the relationship between the obestatin/GPR39 system and gastric cancer progression remains unknown. In the present study, we determined the expression levels of the obestatin/GPR39 system in human gastric adenocarcinomas and explored their potential functional roles. Twenty-eight patients with gastric adenocarcinomas were retrospectively studied, and clinical data were obtained. The role of obestatin/GPR39 in gastric cancer progression was studied *in vitro* using the human gastric adenocarcinoma AGS cell line. Obestatin exogenous administration in these GPR39-bearing cells deregulated the expression of several hallmarks of the epithelial-mesenchymal transition (EMT) and angiogenesis. Moreover, obestatin signaling promoted phenotypic changes via GPR39, increasingly impacting on the cell morphology, proliferation, migration and invasion of these cells. In healthy human stomachs, obestatin expression was observed in the neuroendocrine cells and GPR39 expression was localized mainly in the chief cells of the oxyntic glands. In human gastric adenocarcinomas, no obestatin expression was found; however, an aberrant pattern of GPR39 expression was discovered, correlating to the dedifferentiation of the tumor. Altogether, our data strongly suggest the involvement of the obestatin/GPR39 system in the pathogenesis and/or clinical outcome of human gastric adenocarcinomas and highlight the potential usefulness of GPR39 as a prognostic marker in gastric cancer.

## INTRODUCTION

Gastric cancer is the second leading cause of cancer deaths worldwide, with a frequency that varies greatly across different geographic locations. Despite the decreasing worldwide incidence, gastric cancer accounts for 3% to 10% of all cancer related deaths. Overall, 95% of the stomach cancers are adenocarcinomas, while the remaining cancers are essentially lymphomas and sarcomas [1]. This type of cancer is diagnosed at an advanced stage accompanied by malignant proliferation in most patients, and the prognosis for advanced stage patients is still very poor [2]. A crucial barrier to the development of more effective treatment strategies is the lack of biomarkers for predicting and monitoring the pathological progression of the disease. Many genes and factors, growth factors and signaling targets have been identified as key players underlying tumorigenesis of gastric cancer. Nevertheless, their applications as biomarkers remain debatable given that the prognostic and/or predictive value has not been fully established. As a result, no commonly accepted biomarkers have been established to facilitate the comprehensive management of these patients. Thus, the identification of well-established molecular biomarkers and therapeutic targets will provide a tool to optimize the diagnosis and treatment.

An approach to biomarker identification is the characterization of systems underlying gastric cancer cell proliferation. In this context, obestatin, a 23-amino acid peptide derived from the ghrelin peptide precursor (preproghrelin), was originally identified in the stomach [3], and its receptor, GPR39, was demonstrated to exert a pivotal role in many physiological functions and in multiple diseases, including cancer. Indeed, the GPR39 receptor was found to be overexpressed in primary esophageal squamous cell carcinomas (ESCCs), which was significantly associated with lymph node metastasis and advanced TNM stage, suggesting that GPR39 plays an important tumorigenic role in the development and progression of ESCC [4]. With reference to gastric cancer, the obestatin/GPR39 system was demonstrated to regulate the proliferation of gastric adenocarcinoma cell lines. The molecular mechanisms that determine the obestatin proliferative fate involve the activation of the GPCR scaffolding protein β-arrestin to promote EGFR transactivation via interactions with membrane-localized matrix metalloproteases (MMPs) [5, 6]. GPCR-induced transactivation of EGFR signaling is recognized as a mechanism that regulates normal cellular responses but has also been implicated in pathologies such as cancer [7, 8]. Although the expression of obestatin was described in human gastric neuroendocrine tumors [9, 10], no studies have provided evidence of the obestatin/GPR39 expression in other types of gastric cancer, especially adenocarcinomas, or the functional role of this system in these cancers. Furthermore, when cancers arise from the gastric mucosa, the main site of obestatin production, it is uncertain if they retain the obestatin-producing attributes of their parent mucosa. The fact that obestatin stimulates mitogenesis of gastric cancer cells [5, 6] points to the involvement of this peptide as fuel for gastric cancer cell proliferation. This information led us to postulate the implication of the obestatin/GPR39 system in the development, maintenance and malignancy of gastric cancer. For this purpose, we studied the expression pattern and significance of the obestatin/GPR39 system in human gastric adenocarcinomas. Furthermore, the underlying obestatin/GPR39 mechanism of action was determined using the human gastric adenocarcinoma cell line AGS.

## RESULTS

### Obestatin and GPR39 are expressed in AGS cells

**Figure 1:**
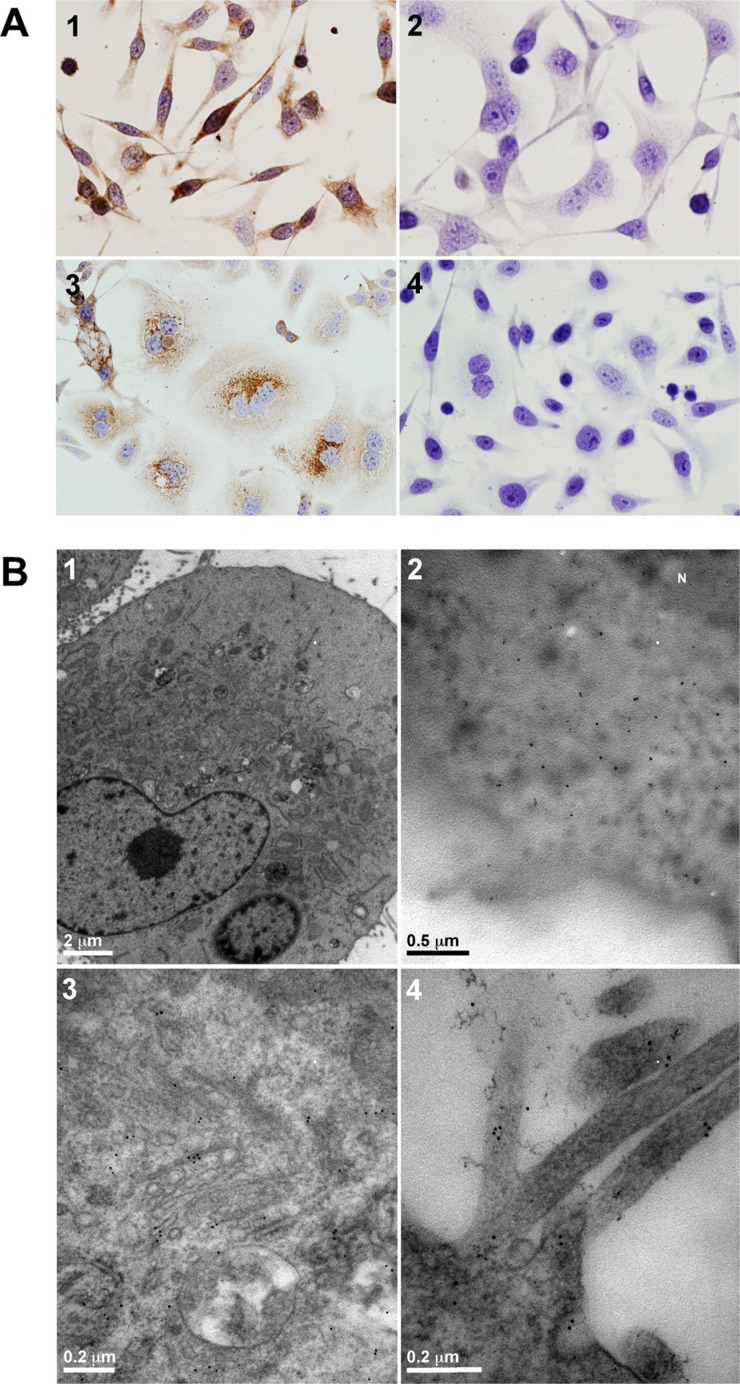
Obestatin and GPR39 are expressed in AGS cells (**A**) Immunocytochemical expression of obestatin and GPR39. 1) Obestatin immunoreactivity was intense and diffuse in the cytoplasm. 2) The preadsorption control was performed applying the primary antibody plus obestatin (10 nmol/mL per control peptide) to positive samples. 3) The expression of GPR39 presented a perinuclear location. 4) The preadsorption control was performed applying the primary antibody plus GPR39 control peptide (10 nmol/mL per control peptide) to positive samples. Images were taken with objective magnification of x20. (**B**) Electron micrograph of obestatin and GPR39 expression. 1) Morphology of the AGS cells in culture. 2) The expression of obestatin in the AGS cells showed a diffuse cytoplasmic pattern. 3) The expression of GPR39 was located in the mature face of the Golgi area. 4) The expression of GPR39 was located in the cell membrane, mainly in the microvilli.

As a model for this study, we selected the gastric adenocarcinoma AGS cell line. This cell line was derived from an untreated human adenocarcinoma of the stomach and retained the same cytological characteristics as the original malignant cells obtained from the patient [11]. As seen in Figure 1A, the expression of the obestatin/GPR39 system was detected by immunocytochemistry. Intense and diffuse obestatin immunostaining was found in the cytoplasm (Figure 1A1), whereas GPR39 was detected in the perinuclear region (Figure 1A3). No immunostaining was found when obestatin or GPR39 antibodies were preadsorbed with homologous peptides (Figure 1A2 and 1A4, respectively). Moreover, an electron micrograph of obestatin expression showed a diffuse cytoplasmic pattern (Figure 1B2), while GPR39 was located in the mature face of the Golgi area, as well as a clear expression in the cell membrane, mainly located in the microvilli (Figure 1B3 and 1B4, respectively).

### Obestatin induces proliferation in an autocrine/paracrine manner in AGS cells

**Figure 2:**
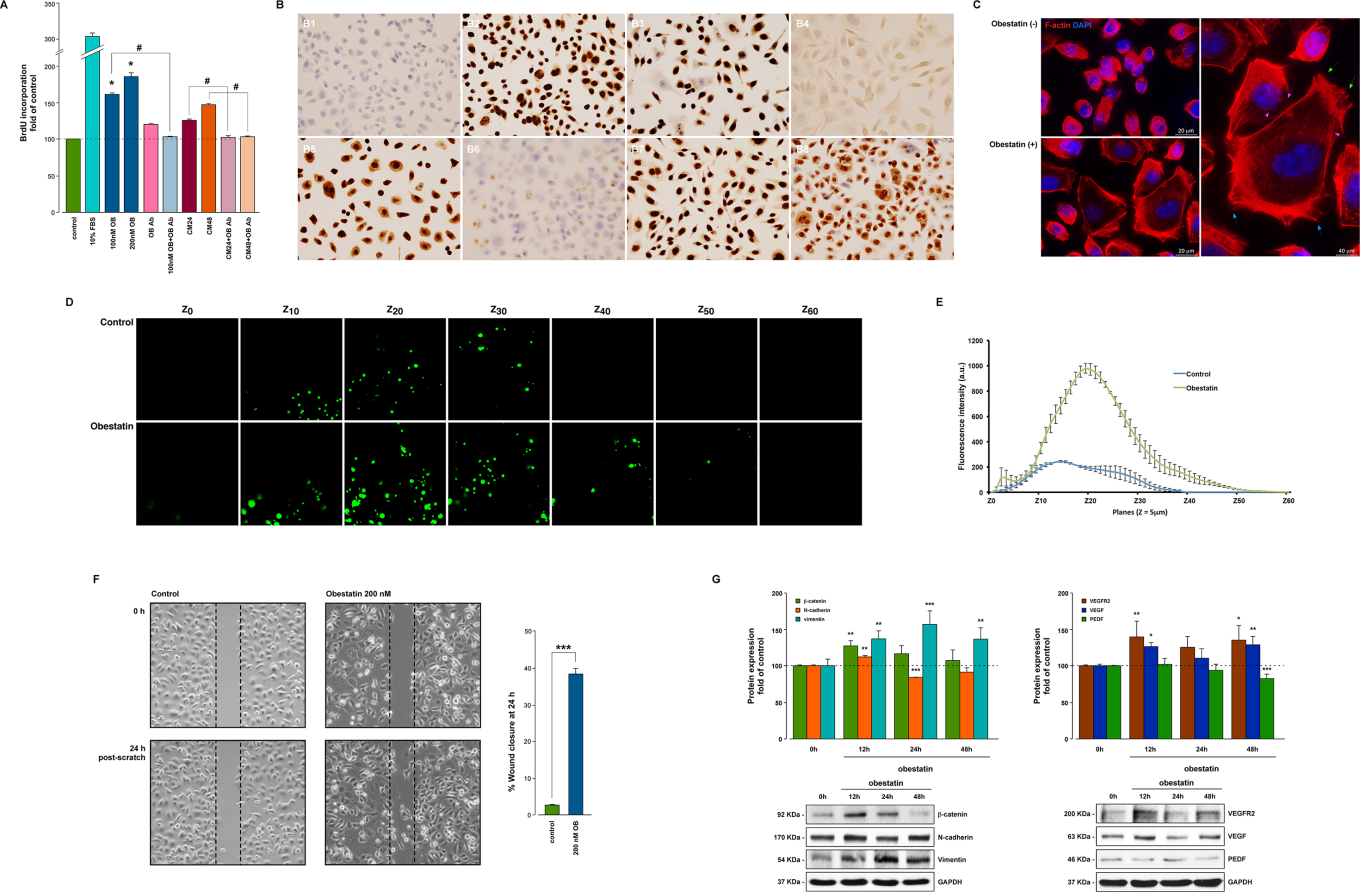
Obestatin promotes proliferation, cell mobility and invasion in AGS cells (**A**) Mitogenic effect of obestatin in the AGS cells. AGS cells were treated with FBS (10%, v/v), OB (100 nM and 200 nM), OB-Ab (10 μg/mL), OB (100 nM) plus OB-Ab, CM24, CM48, CM24 + OB-Ab and CM48 + OB-Ab, and cell proliferation was evaluated after 48 h by means of BrdU incorporation. The data were expressed as a percentage of the basal proliferation of the untreated cells (Mean ± SEM). The asterisk (*) denotes *P* < 0.05 when comparing the OB treated group with the control untreated group; the dagger (#) denotes *P* < 0.05 when comparing the OB or CM plus OB Ab group with the OB or CM treated group. (**B**) Immunocytochemical expression of Ki67 in AGS cells after 24 hour of proliferation. B1) Control without treatment. B2) 10% FBS. B3) 100nM OB. B4) 100nM OB plus OB-Ab (10 μg/mL). B5) CM24. B6) CM24 plus OB-Ab. B7) CM48. B8) CM48 plus OB-Ab. Objective magnification x20. (**C**) Effect of obestatin on the cytoskeleton reorganization in AGS cells. The AGS cells were stimulated with 200 nM OB for 24 h. Cells were stained with Phalloidin CruzFluor-594 (red) to visualize F-actin and DAPI (blue) to visualize the nucleus. Scale bar 20 μm. The image at the right represents a higher magnification view of the obestatin treated cells (scale bar 40 μm). Obestatin treatment induced cellular elongation with the formation of filopodia-like structures (blue arrows), lamellipodia-like structures (green arrows) and the development of stress fibers (pink arrows). Images are representative for at least three independent experiments. (**D**) Obestatin promotes invasion in AGS cells. Sequential confocal microscopy sections scanned every 5-μm from the membrane to the top of the Matrigel in an inverted invasion assay. Obestatin (200 nM) was used as a chemoattractant. (**E**) Mean fluorescence intensity (a.u.) quantified at the indicated sequential confocal sections. Bars, SEM; ***P < 0.001. (**F**) Migration of AGS cells promoted by obestatin. The AGS cells were treated or not with obestatin (200 nM). The wound was calculated by tracing along the border of the scratch using ImageJ64 analysis software and the following equation: %wound closure = [[wound area (0 h)-wound area (x h)] / wound area (0 h)] × 100. The asterisk (***) denotes *P* < 0.001 when comparing the treated with the untreated control group. (**G**) Immunoblot analysis of the EMT and the pro-angiogenic activation of obestatin in AGS cells. The AGS cells were stimulated with obestatin (200 nM) for 12, 24, and 48 h and the blots were incubated with the corresponding antibodies to N-cadherin, β-catenin, vimentin, VEGF, VEGF-R2 and PEDF. The protein expression was normalized relative to GAPDH. The data were expressed as mean ± SE obtained from intensity scans of six independent experiments. The asterisk (*, **, ***) denotes *P* < 0.05, *P* < 0.01 and *P* < 0.001 when comparing the treated with the untreated control group.

As Figure 2A shows, obestatin (100 nM and 200 nM) exerted a mitogenic effect in the AGS cell line (162 ± 1% and 181 ± 2%, respectively) by means of BrdU incorporation. The neutralization of obestatin (100 nM) by anti-obestatin Ab preincubation diminished the BrdU incorporation to control levels. The autocrine/paracrine role of obestatin was tested by a combination of serum-free conditioned medium from AGS cells obtained at 24 and 48 h (CM24 and CM48, respectively) with neutralizing obestatin antibody. The treatment of AGS cells with CM24 caused an increase in proliferation, which was completely abolished by CM-24 h + anti-obestatin Ab treatment. Similar results were obtained with the CM48 treatment, but with a higher impact. Likewise, the immunocytochemical analysis of the proliferation marker Ki67 [12] confirmed the proliferative activity of obestatin (Figure 2B). The Ki67-immunopositive cells after obestatin treatment (100 nM, 24 h; Figure 2B3) were similar to those obtained with FBS (Figure 2B2). Anti-obestatin Ab clearly reduced the Ki67 expression levels with respect to standard conditions (Figure 2B4). Furthermore, the presence of the anti-obestatin Ab reduced the Ki67 expression of AGS cells treated with CM24 (Figure 2B6 and 2B5, respectively), whereas Ab treatment partly decreased Ki67 expression in CM48-treated cells (Figure 2B8 and 2B7, respectively). This discrepancy with CM24 might be due to the numerous factors secreted by the cells, which were present in CM after 48 h.

### Obestatin promotes cell mobility and invasion via EMT and cytoskeleton remodeling in AGS cells

AGS cells are organized in clusters of polygon-shaped cells, few actin short stress fibers, and no lamellipodia with their cobblestone-like phenotype. These actin filaments in the form of stress fibers and the thin network formed at the edges could be depolymerized by the removal of serum, although the phenomenon was reversible when the cells were returned back to serum containing medium [13]. We then analyzed the effect of obestatin on the morphology and cytoskeleton in serum-free medium to avoid interference (Figure 2C). Under these conditions, obestatin treatment (200 nM, 24 h) promoted the dissociation of the cell clusters and induced cellular elongation with the formation of filopodia-like structures (blue arrows) and lamellipodia-like structures (green arrows) typical of motile cells. Obestatin also induced strong actin polymerization including the development of stress fibers (pink arrows). Obestatin-treated cells presented the scattering/hummingbird-like phenotype previously reported in the case of *Helicobacter pylori* infection, mimicking an EMT [14].

We analyzed whether obestatin was driving cell invasion using a 3-dimensional (3D) culture assay. For this purpose, the inverted version of the classical Boyden chamber invasion assay was used [15]. As shown in Figure 2D, the AGS cells were able to migrate through the membrane, mimicking basement membrane invasion and invade into the Matrigel as an extracellular matrix when obestatin was applied on top of the Matrigel as a chemoattractant (200 nM). The extent of cell invasion was quantified by measuring the fluorescence intensity at each confocal section every 5 μm from the membrane, and the differences between the untreated and treated cells were statistically significant (*P* < 0.001; Figure 2E). Aditionally, the wound-healing assay showed that the obestatin-treated cells exhibited a significant increase in their migration capability when compared with the control (*P* < 0.001; Figure 2F).

EMT is proposed to regulate the acquisition of migratory and invasive capability, which is a crucial mechanism in the initial steps in of metastatic progression [16]. Because obestatin increased migration and invasion, we assessed obestatin's influence on EMT by examining the expression of E-cadherin, β-catenin, vimentin, and N-cadherin (Figure 2G). Obestatin treatment (200 nM) increased β-catenin (active form) and N-cadherin levels after 12 h (127 ± 7% and 112 ± 3%, respectively), whereas vimentin levels showed an intense augmentation along the tested period of time with a maximum at 24 h (158 ± 19%). E-cadherin was not detected in the AGS cells, at least at the limits of immunoblot detection (data not shown). Indeed, this cell line harbored an E-cadherin mutation, leading to a truncated form of the protein that is not expressed [17]. Regarding the VEGF/VEGF-R2 system, obestatin increased the VEGF and VEGF-R2 levels at 12 h and 48 h (VEGF: 126 ± 5% and 128 ± 12%; VEGF-R2: 140 ± 21% and 134±20%, respectively; Figure 2G), whereas it decreased the anti-angiogenic factor PEDF levels with a significant minimum at 48 h (72 ± 7%).

### Obestatin exerts its actions through the GPR39 receptor in AGS cells

**Figure 3:**
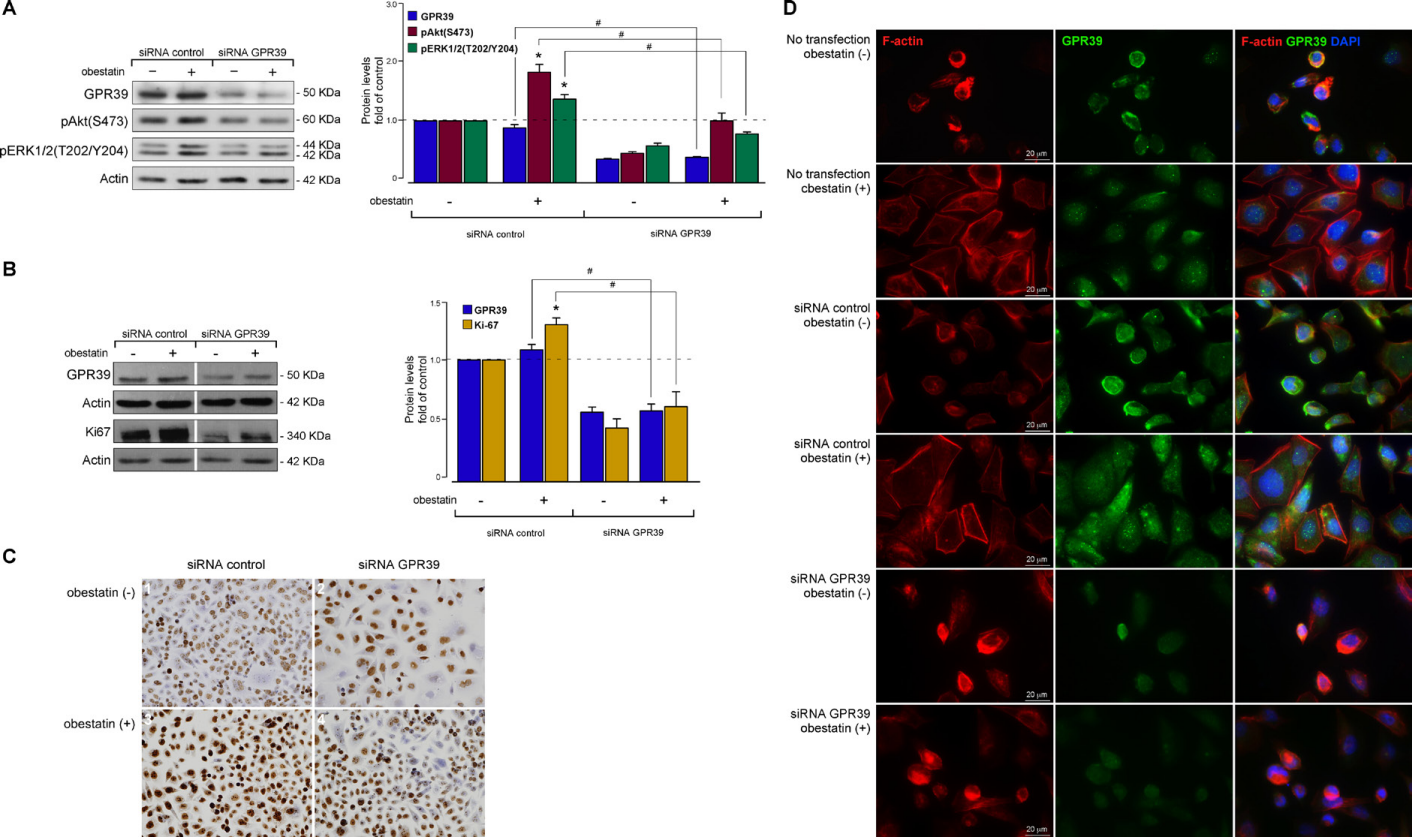
Obestatin exerts its actions through the GPR39 receptor in AGS cells (**A**) Effect of GPR39 knockdown by siRNA on obestatin-activated pAkt(S473) and pERK1/2(T202/Y204) in AGS cells. The AGS cells were transfected with GPR39 siRNA prior to obestatin treatment (200 nM, 10 min). GPR39 was expressed as a fold of its level to control siRNA-transfected cells (*n* = 3). Activation of Akt [pAkt(S473)] and ERK1/2 [pERK1/2(T202/Y204)] was expressed relative to the control siRNA-transfected cells. (**B**) Effect of SiRNA depletion of GPR39 on obestatin-activated Ki67 expression in AGS cells. The AGS cells were transfected with GPR39 siRNA prior to obestatin treatment (200 nM, 24 h). Expression of Ki67 was denoted as a fold of the respective levels in control siRNA-transfected cells (*n* = 3). Equal amounts of protein in each sample were used to assess the expression of GPR39 by western blotting. The level of proteins was expressed as fold change relative to the control siRNA-transfected cells (mean ± SE). The protein expression was normalized relative to actin. The data were expressed as mean ± SE obtained from intensity scans of independent experiments. The asterisk (*) denotes *P* < 0.05 when comparing the treated control siRNA group with the control siRNA group; the dagger (#) denotes *P* < 0.05 when comparing the GPR39 siRNA group with the control siRNA group. (**C**) Effect of GPR39 knockdown by siRNA on the immunocytochemical expression of obestatin-activated Ki67 in AGS cells. The AGS cells were transfected with GPR39 siRNA prior to obestatin treatment (200 nM, 24 h). 1) siRNA control without treatment. 2) siRNA GPR39 without treatment. 3) siRNA control plus OB. 4) siRNA GPR39 plus OB. Images were taken with objective magnification x20. (**D**) Effect of GPR39 knockdown by siRNA on the cytoskeleton reorganization in AGS cells. The AGS cells were transfected or not with GPR39 siRNA prior to obestatin treatment (200 nM, 24 h). Cells were stained with Phalloidin CruzFluor-594 (red), anti-GPR39 antibody (green), and DAPI (blue). Scale bar 20 μm. Images are representative for at least three independent experiments.

First, the effect of acute GPR39 deficiency on obestatin signaling was determined by treatment of AGS cells with a GPR39 small interfering RNA (GPR39 siRNA). Under these conditions, the constructs decreased GPR39 expression by 65 ± 2% (Figure 3A). In the presence of an si-control, the obestatin-activated Akt(S473) and ERK1/2(T202/Y204) phosphorylations were identical to the levels observed with the untransfected cells (data not shown). The silencing of GPR39 decreased subsequent pAkt(S473) and pERK1/2(T202/Y204) with respect to the control siRNA by 43 ± 3% and 40 ± 4% upon treatment of obestatin (200 nM, 10 min; Figure 3A), respectively. Second, the effect of acute GPR39 deficiency on Ki67 expression was evaluated. Under these conditions, the constructs decreased GPR39 expression by 53 ± 7% (Figure 3B). The GPR39 knockdown decreased obestatin-induced Ki67 expression by 70 ± 4% (Figure 3B) with respect to the si-control. Immunocytochemistry confirmed decreased levels of Ki67 in GPR39 knockdown cells compared with control cells (Figure 3C).

The above results implicated the obestatin/GPR39 system in EMT and cell motility, possibly by cytoskeletal modulation. Consistent with a role in actin polymerization, the GPR39 knockdown cells exhibited depolymerization and redistribution of the cellular F-actin, diminishing lamellipodia formation compared to the control cells after obestatin treatment (Figure 3D). Taken together, these results suggest that the obestatin/GPR39 system induces cytoskeleton remodeling to facilitate AGS cell migration and invasion.

### GPR39 and obestatin expression in human healthy stomach tissue

**Figure 4:**
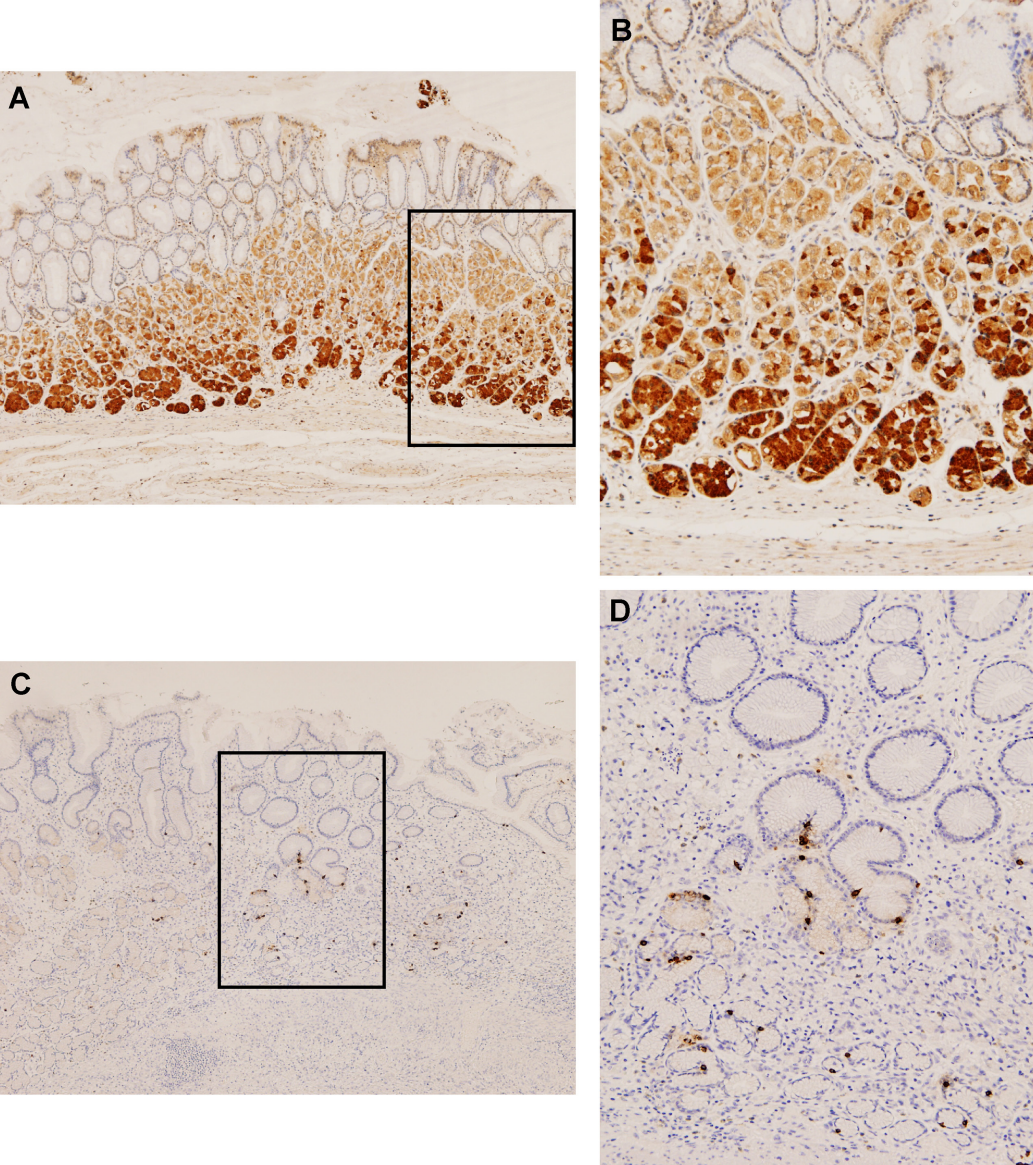
GPR39 and obestatin are expressed in human healthy stomach tissue Immunohistochemical expression of GPR39 and obestatin in normal stomach. (**A**) There was no GPR39 expression in the mucosal cells of the gastric pits. A weak positivity was observed in parietal cells, whereas an intense immunostaining was present in the chief cells (x4). (**B**) Higher magnification view (x10) of the GPR39 immunostaning in the chief cells. (**C**) Obestatin immunoreactivity was only observed in neuroendocrine cells of oxyntic glands (x4). (**D**) At higher magnification, obestatin producing cells were recognized as small round or spindle cells with brownish staining in the cytoplasm (x10).

As shown in Figure 4, no GPR39 expression was detected in the mucosal cells of the gastric pits. Weak positivity was observed in parietal cells, whereas intense immunostaining was present in the chief cells located at the base of the oxyntic glands (Figure 4A and 4B). Regarding obestatin expression, the obestatin-immunoreactive (IR) endocrine cells were localized from the neck to the base of the gastric glands in the oxyntic mucosa (Figure 4C and 4D). These cells were small and roundish and showed typical morphology of closed-type endocrine cells, which were devoid of elongation contacting the lumen [18].

### GPR39, obestatin and Ki67 expression in human gastric adenocarcinomas

**Figure 5:**
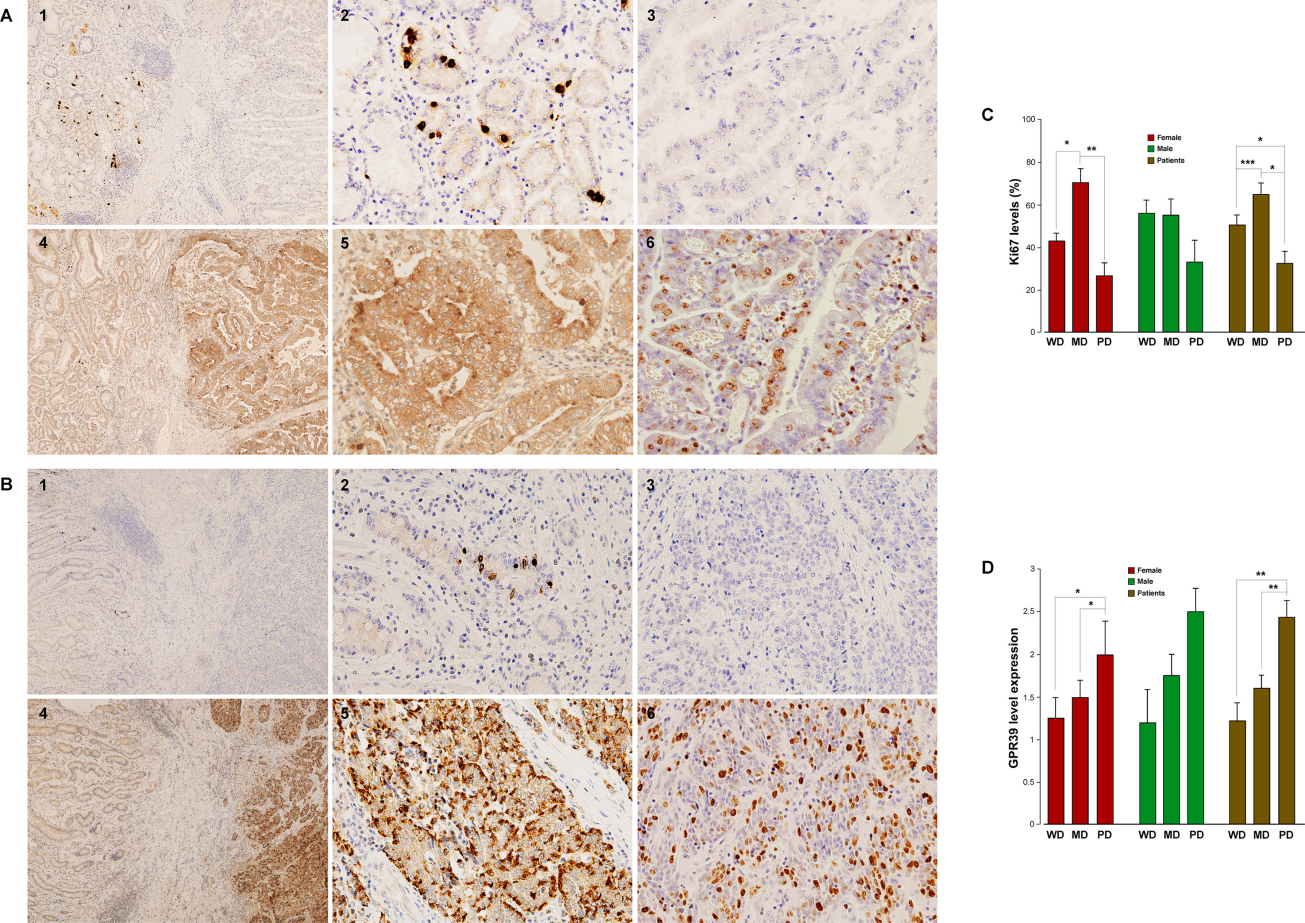
GPR39, obestatin and Ki67 expression in human gastric adenocarcinomas (**A**) Immunohistochemical expression of obestatin, GPR39, and Ki67 in a well-differentiated gastric adenocarcinoma. 1) Obestatin expression was negative in all of the well-differentiated adenocarcinoma (right), whereas normal gastric mucosa (left) showed immunoreactivity only in the neuroendocrine cells, (x4). 2) Higher magnification view of the intense obestatin positivity in neuroendocrine cells of the normal gastric mucosa (x20). 3) Obestatin expression was negative in the well-differentiated gastric adenocarcinoma (x20). 4) GPR39 expression was positive in the tumor, whereas no immunostaining was found in the normal gastric mucosa (x4). 5) Higher magnification view of GPR39 positivity (x20). 6) Moderate Ki67 expression in this well-differentiated adenocarcinoma. (**B**) Immunohistochemical expression of obestatin and GPR39 in a poorly differentiated gastric adenocarcinoma. 1) Obestatin expression was negative in all of the gastric adenocarcinomas studied. Positivity for obestatin was only observed in neuroendocrine cells of gastric mucosa (x4). 2) Higher magnification view of obestatin positivity in the neuroendocrine cells (x20). 3) Obestatin was negative in the poorly differentiated gastric adenocarcinoma (x20). 4) GPR39 expression was positive in the tumor, whereas no immunostaining was found in the mucosa (x4). 5) Higher magnification view of GPR39 positivity (x20). 6) Moderate Ki67 expression in this poorly differentiated adenocarcinoma (x20). (**C**) Graphical representation of the Ki67 PI in the studied gastric adenocarcinomas. The asterisk (*, **, ***) denotes *P* < 0.05, *P* < 0.01 and *P* < 0.001 when comparing groups. (**D**) Graphical representation of the GPR39 levels of expression in the studied gastric adenocarcinomas. The asterisk (*, **) denotes *P* < 0.05 and *P* < 0.01 when comparing groups.

The characteristics of the patient samples and the results obtained in the gastric adenocarcinomas studied are summarized in Table 1. Figure 5 shows the results obtained for obestatin, GPR39, and Ki67 in the samples of human gastric adenocarcinomas, represented by a well-differentiated (WD) and a poorly differentiated (PD) adenocarcinoma (Figure 5A and 5B, respectively). In these samples, obestatin (Figure 5A1 and 5B1) was negative in all of the gastric adenocarcinomas studied (Figure 5A3 and 5B3), whereas intense staining was found in the neuroendocrine cells of the healthy gastric mucosa (Figure 5A2 and 5B2), although it was negative in the rest of the oxyntic mucosa. By contrast, GPR39 positivity was present in all of the tumors with varying intensity according to the classification of the samples. Lower expression was found in WD tumors (mostly 1+), and the highest expression was observed in the PD adenocarcinomas (2+/3+ or 3+, predominantly). The Golgi staining was more intense, in general, for the WD adenocarcinomas (Figure 5A4 and 5A5), was diminished for the moderately differentiated (MD) and was not detectable in the majority of the analyzed PD adenocarcinomas, although three of them presented high positivity in the Golgi area (Figure 5B4 and 5B5).

**Table 1:** Summary of the characteristics and the obestatin, GPR39 and Ki67 immunostain results of the gastric adenocarcinomas studied

Well-differentiated							
Lauren classification	Age	Sex	Size (cm)	TNM staging	obestatin	GPR39	Ki67 (%)
Intestinal	72	F	3.0	pT4N2M1	NM-NC	2+, homogeneous	39
Intestinal	81	F	6.8	pT2bN1	0	1+, homogeneous	44
Intestinal	67	M	3.5	pT4N3M1	NM-NC	1+, heterogeneous	48
Intestinal	53	M	5.0	pT3N1	NM-NC	1+, heterogeneous	59
Intestinal	81	F	4.3	pT2	NM-NC	1+, heterogeneous	53
Intestinal	83	M	1.7	pT1N0	NM-NC	0, homogeneous	66
Intestinal	69	F	4.3	pT4	0	1+, homogeneous	37
Intestinal	76	M	4.3	pT3N1	NM-NC	2+, heterogeneous	37
Intestinal	81	M	7.5	pT3N3	NM-NC	2+	71
**Moderately differentiated**							
Intestinal	88	F	6.5	pT3	0	1+	53
Intestinal, with SRC	84	F	7.0	pT4N3M1	NM-NC	1+	79
Intestinal	68	F	6.8	pT4N3	0	2+, heterogeneous	72
Intestinal	53	F	2.0	pT4N1	0	1+	49
Intestinal	40	M	2.1	pT4	0	2+	69
Intestinal	72	M	7.5	pT4N3	NM-NC	2+	37
Intestinal	49	F	4.2	pT3N1	0	2+	93
Intestinal	82	F	7.5	pT4N3	0	2+, heterogeneous	81
Intestinal	61	M	5.1	pT4N2	NM-NC	2+	51
Intestinal	85	M	7.5	pT4N0	NM-NC	1+	68
**Poorly differentiated**							
Intestinal	76	M	7.0	pT3N3Mx	0	2+	21
Diffuse, with SRC	58	F	1.6	pT2Nx	NM-NC	2+, SRC negative	20
Mixed	67	M	5.7	pT3N3	NM-NC	2+	19
Mixed, with SRC (< 50%)	80	F	7.5	pT3N0	NM-NC	3+	37
Diffuse	78	F	6.0	pT4N0	NM-NC	2+	23
Intestinal	85	M	6.5	pT2N1	NM-NC	3+	63
Mixed, with SRC	79	F	4.9	pT4N1	0	3+	31
Intestinal	64	M	4.5	pT4N2	0	3+	30
Diffuse, with SRC	62	F	2.3	pT2N1	0	2+, heterogeneous	47

F: female; M: male; NM-NC: Normal mucosa neuroendocrine cells; SRC: signet ring cells.

Ki67 immunohistochemistry using an MIB-1 antibody was used to evaluate the proliferative activities. Ki67 immunohistochemistry produced a mean Ki67 proliferative index (PI) of 50.4% ± 1.62 in the WD, 65.2% ± 1.9 in MD and 29.5% ± 1.8 in PD adenocarcinomas (Figure 5C: WD/MD, ***P < 0.001; MD/PD, *P < 0.05; and WD/PD, *P < 0.05). These results support previous data demonstrating that low Ki67 PI might lead to an unfavorable prognosis as it has been correlated to poorly differentiated histology, an advanced T stage, lymph node metastasis, and greater expression of EMT-related proteins [19]. Strong and enigmatic male dominance has been described in the incidence of gastric cancer with a male-to-female ratio of approximately 2:1, and these differences may be related to the protective effect of estrogens, especially for the intestinal-type adenocarcinomas [20]. Regarding Ki67 expression, we observed the following results for males: WD, 56.2% ± 6.2; MD, 56.2% ± 7.6; and PD: 33.3% ± 10.2. For female patients, the results were as follows: WD, 45.5% ± 3.4; MD, 71.2% ± 6.9; and PD, 31.6% ± 4.9, with statistically significant differences between WD/MD, and MD/PD (*P < 0.05 and **P < 0.01, respectively). However, the results for GPR39 expression correlated with the dedifferentiation of the tumor, with increased expression from WD (1.22 a.u. ± 0.2), MD (1.60 a.u. ± 0.2), to PD (2.44 a.u. ± 0.2) (Figure 5D: WD/PD, **P < 0.01; and MD/PD, **P < 0.01; Figure 5D). The expression levels organized by sex followed the same tendency, although the differences between the groups were statistically significant only in females (WD/PD, *P < 0.05; and MD/PD, *P < 0.05). These patterns of expression are summarized in Figure 5C and 5D, and Table 2 shows the correlations in these samples.

**Table 2:** Pearson correlation of the data

	Age	Sex	Size	Differentiation	GPR39 expression	Ki67 expression
**Age**	-	0.640	0.002**	0.797	0.682	0.823
**Sex**	0.09	-	0.812	0.651	0.902	0.780
**Size**	0.57	−0.05	-	0.477	0.217	0.633
**Differentiation**	−0.05	0.9	0.14	-	0.000***	0.049*
**GPR39 expression**	−0.08	−0.02	0.24	0.66	-	0.182
**Ki67 expression**	−0.04	0.06	0.09	−0.37	−0.26	-

Correlation coefficient (*r*) below and *P*-values above the diagonal.

* means significant at the 0.05 level (2-tailed);

** means significant at the 0.01 level (2-tailed);

*** means significant at the 0.001 level (2-tailed). *N* = 28.

## DISCUSSION

In this study, we determined the role of the obestatin/GPR39 system in regulating motility, EMT, and invasion of adenocarcinoma cells. First, we found that obestatin promoted cell mobility and invasion by inducing EMT and cytoskeleton remodeling in AGS cells.

Second, we showed that GPR39 was present in the chief cells located at the base of the oxyntic glands, while obestatin was identified in the neuroendocrine cells of the oxyntic glands. Third, the GPR39 pattern correlated with the dedifferentiation of gastric adenocarcinomas, and the obestatin expression was negative. We propose that the obestatin/GPR39 system displays an enhancer role in the development and progression of gastric adenocarcinoma.

The obestatin/GPR39 system has important regulatory roles in controlling proliferation and differentiation through a fine modulation of key players in these cellular pathways. Specifically, the obestatin/GPR39 system has been described to act as a pro-proliferative signal in gastric cancer cells, namely, KATO-III and AGS [5, 6]. For simplicity, and similar to the human gastric adenocarcinomas, we used the AGS cell line, which expresses the obestatin/GPR39 system and retains the characteristics of the parent tumor. In this study, our findings implicated an autocrine/paracrine regulation of cell proliferation, which activated Ki67 expression. Furthermore, this study shows that the obestatin/GPR39 system is a key regulator of EMT. This conclusion is based on the observation that obestatin induced a repertoire of biochemical (increased β-catenin, N-cadherin, and vimentin) and morphological (increased formation of lamellipodia) changes, that are characteristic of EMT. Thus, cytoskeleton reorganization induced by obestatin/GPR39, shifting its epithelial phenotype to an invasive-like one, facilitated tumor migration, invasion, and metastasis. Recent studies have implicated mTORC1 and mTORC2 as key regulators of EMT [21]. Indeed, GPR39 activates mTOR signaling via the β-arrestin/MMP/EGFR/Akt pathway [6]. Additionally, obestatin was shown to increase the expression of VEGF and its receptor isoform VEGFR2 in these cells. Conversely, PEDF expression was down-regulated. Notably, the VEGF/VEGFR2 system is primarily responsible for both normal and tumor-related angiogenesis [22]. This finding implies that VEGF, which is produced by obestatin-stimulated cells, provides the needed vascularization of the developing tumor. This postulate is further supported by the down-regulation of the anti-angiogenic factor PEDF [23].

In healthy human stomachs, positive expression of obestatin was observed in the neuroendocrine cells, while GPR39 expression was identified in the chief cells of the oxyntic glands. Therefore, the idea of an autocrine positive feedback of obestatin was ruled out; however, an endocrine mechanism by which obestatin accesses its own transmembrane receptor to optimally mediate receptor-ligand interactions is possible. Taking into account the GPR39 location, obestatin may regulate pepsinogen secretion in the chief cells, an important fact for the digestive process in the stomach (submitted manuscript peer review).

A particularly interesting result is that chief cells expressed GPR39 together with the expression found in human adenocarcinomas. This fact might relate this cell type to the origin of the gastric adenocarcinoma. Indeed, the potential correlation between the chief cells and gastric cancer has been proposed [24, 25]. These studies provided direct evidence by lineage tracing that under pathophysiological loss of acid-secreting parietal cells, the chief cell lineage can itself transdifferentiate into a mucous cell metaplasia, designated spasmolytic polypeptide expressing metaplasia, a precancerous lesion in stomach. Particularly in the presence of inflammation, this metaplastic lineage can regain proliferative capacity and, in humans, may also further differentiate into intestinal metaplasia. Thus, mature gastric chief cells have the ability to act as cryptic progenitors and reacquire proliferative capacity within the context of mucosal injury and inflammation.

Of major clinical significance was the observation that the GPR39 expression found in gastric adenocarcinomas correlated with the dedifferentiation of the tumor. The histologic type is important for estimating tumor progression and the outcomes of patients with gastric carcinoma, as it represents an independent prognostic factor among the pathological variables of the tumor [26]. In that sense, the correlation found for GPR39 and the histologic type endorse the use of this receptor as a marker of tumor progression.

In conclusion, the present work addresses the role of the obestatin/GPR39 system in regulating motility, EMT, and invasion of gastric adenocarcinoma cells. More importantly, the levels of GPR39 expression in these tumors provide the rationale for including GPR39 as a prognostic marker of human gastric adenocarcinomas.

## MATERIALS AND METHODS

### Materials

The antibodies and peptides used are listed in Table 3. All other chemical reagents were from Sigma Chemical (St. Louis, MO).

**Table 3:** Materials

Materials	Use	Dilution	Code	Manufacturer
Anti-Human GPR39 peptide antibody	IC IEM	1:500 1:50	GPR392-A	Alpha Diagnostic International
Anti-Human GPR39 (C-terminal region) antibody	IH	1:500	SAB4200185	Sigma Chemical
Anti-GPCR GPR39 antibody	WB IF	1:1000 1:500	ab39227	Abcam
Anti-Mouse/rat/human Obestatin peptide antibody	IC, IH IEM	1:500 1:50	OBSN11-A	Alpha Diagnostic International
Obestatin (Human, Monkey) Antibody	NA	10 μg/mL	G-031-92	Phoenix Pharmaceuticals
Anti-Human Ki-67 Antigen Clone MIB-1	IC, IH	Ready to use	M724001	Dako
Anti-Ki67 antibody	WB	1:1000	ab15580	Abcam
Phospho-p44/42 MAPK (Erk1/2) (Thr202/Tyr204) Antibody	WB	1:2000	9101S	Cell Signaling Technology
Phospho-Akt (Ser473) Antibody	WB	1:2000	9271S	Cell Signaling Technology
Anti-Actin antibody – Loading Control	WB	1:4000	ab1801	Abcam
Peroxidase-AffiniPure Goat Anti-Rabbit IgG (H+L)	WB	1:10000	111-035-003	Jackson ImmunoResearch Europe
Anti-Active beta catenin Antibody	WB	1:1000	05-665	Millipore
Anti-N-Cadherin Antibody	WB	1:1000	ab76011	Abcam
Anti-Vimentin Antibody	WB	1:1000	ab92547	Abcam
Anti-Flk1 (C-1158) Antibody (VEGFR2 antibody)	WB	1:500	sc-504	Santa Cruz
Anti-VEGF (A-20) Antibody	WB	1:500	sc-152	Santa Cruz
Anti-PEDF (H-125) Antibody	WB	1:500	sc-25594	Santa Cruz
Anti-GAPDH Antibody	WB	1:1000	ab9485	Abcam
Alexa Fluor 488 Antibody	IF	1:1000	ab150089	Abcam
Phalloidin CruzFluor^™^-594	IF	1:1000	sc-363795	Santa Cruz Biotechnology
Human obestatin	-	-	471–97	California Peptide Research
Human GPR39 control antigen peptide	IC	1:5*	GPR392-P	Alpha Diagnostic International
Human GPR39 control antigen peptide	WB	1:5*	ab39283	Abcam
Mouse/rat/human obestatin control peptide	IC	1:5*	OBSN11-P	Alpha Diagnostic International

Relation of the materials used in the different analyses performed in this work. Abbreviations: IC: immunocytochemistry; IEM: immunoelectron microscopy; IH: immunohistochemistry; WB: western blot; NA: neutralization assay; IF: immunofluorescence; RT: room temperature; O/N: overnight. *10 nmol/mL

### Cell culture

The human gastric adenocarcinoma AGS cell line was cultured as described by the supplier (ECACC, Wiltshire, UK). Briefly, AGS cells were seeded in 100-mm dishes and cultured in Ham's F12 medium supplemented with 10% (v/v) fetal bovine serum (FBS), 100 U/mL penicillin G, 100 U/mL streptomycin sulfate and 2.5 mM L-glutamine with 5% CO_2_ at 37°C. This cell line was always used with passages lower than 15 in all of the experiments. The AGS cells were bought in May 2013 from Sigma-Aldrich, which obtained the cells from ECACC (Product code: 89090402; Lot Number: 06G018). ECACC performed the following tests: 1) cell count, viability and confluency of cells on resuscitation after being frozen (SOP ECC108; 18/08/2006); 2) detection of mycoplasma by PCR using mycoplasma-specific PCR primers validated by ECACC (SOP ECC73; 18/08/2006); 3) detection of mycoplasma using a Vero indicator cell line and Hoechst 33258 fluorescent detection system (SOP ECC123; 18/08/2006); 4) speciation by isoenzyme analysis where the migration of lactate dehydrogenase (LD), glucose-6-dehydrogenase (G6PD), nucleoside phosphorylase (NP) and malate dehydrogenase (MD) of the test cell line is assessed by electrophoresis (SOP/ECACC/006; 29/08/2006); 5) authentication of human cell lines by STR profiling (SOP ECC86; 29/08/2006), where the human cell lines were authenticated using the AmpFISTR^®^ SGM Plus^®^ PCR amplification kit and the ABI Prism 3730 genetic analyzer, and the SGM Plus^®^ kit amplified 10 STR loci plus the sex markers; and 6) sterility testing of Cell Banks (SOP ECC92; 22/08/2006).

### Immunocytochemistry detection of GPR39 and obestatin

The AGS cell samples were processed using standard procedures [27]. The slides were consecutively incubated with 1) anti-GPR39 or anti-obestatin in Dako ChemMate antibody diluent (Dako, Glostrup, DK); 2) EnVision™ peroxidase rabbit (Dako, Carpinteria, CA, US), which was used as the detection system; and 3) 3,3′-diaminobenzidine-tetrahydrochloride (Dako Liquid DAB + Substrate-chromogen system). The cells were faintly counterstained with Harris hematoxylin solution (HHS). The preadsorption control was performed by applying the primary antibody plus obestatin or the GPR39 control peptide to the positive samples.

### Immunocytochemistry detection of Ki67

The AGS cells were cultured at a density of 8 × 10^3^ cells per well in the culture medium described above on 8-well Nunc^®^ Lab-Tek^®^ II chamber slides covered with CC2 glass slide coverslips. After 2 days, the medium was renewed, and the cells were cultured in serum-free medium (300 μL) for 24 h. The cells were then treated with FBS (10% v/v), human obestatin (100 nM; OB), anti-obestatin Ab (10 μg/mL; OB-Ab), OB (100 nM) plus OB-Ab (10 μg/mL), serum-free conditioned medium (CM) from AGS cells obtained after 24 and 48 h (CM24 and CM48, respectively), CM24 plus Ob-Ab (10 μg/mL) and CM48 plus Ob-Ab (10 μg/mL) in fresh Ham's F12 (300 μL). For GPR39-siRNA depleted AGS cells, the cells were transfected using Lipofectamine 2000 (Invitrogen; CA, US). Serum-starved cells were stimulated or not with obestatin (200 nM, 24 h) at 37°C. After 24 h, the intact cells were fixed in 96% ethanol for 1 h. The immunocytochemical technique was performed as described above using the FLEX primary antibody MIB-1. In all cases, triplicate dishes were used for each experiment point.

### Immunoelectron microscopy

The AGS cells were fixed (2–4 h) in a solution of 2% paraformaldehyde and 0.2% glutaraldehyde in 0.1 M sodium phosphate buffer (PBS, pH 7.4). After washing with PBS, the specimens were dehydrated in a graded ethanol series and embedded in LR White (The London Resin Co, Basingstoke, UK). Polymerization was performed at 60°C for 18 h, avoiding contact between the resin and oxygen. Ultrathin sections (60 nm thick) were collected on uncoated 700-mesh nickel grids. Labeling procedures were performed at room temperature (RT). The procedure comprised the following steps: 1) 0.5% ovalbumin (v/v) and 0.05% Tween 20 (v/v) in PBS (Solution A; 2 × 5 min); 2) anti-GPR39 or anti-obestatin antibody in the same buffer as used in step 1 (O/N, RT); 3) Solution A (1 × 10 min); 4) 0.5% ovalbumin (v/v) and 0.05% Tween 20 (v/v) in Tris buffer saline (TBS, 0.02 M Tris-HCl, 0.65 M NaCl) (Solution B; 2 × 10 min); 5) goat anti-rabbit (GAR) labeled with 10 nm (GPR39) or 20 nm (obestatin) colloidal gold (British BioCell International, Cardiff, UK), both diluted at 1:20 in Solution B (1 × 1 h); 6) standard saline citrate (SSC; SSC: 0.15 M NaCl, 0.015 M sodium citrate, pH 7.0; 1 × 5 min); 7) distilled water (1 × 5 min); 8) 2% neutral uranyl acetate (1 × 2 min); and 9) distilled water (2 × 5 min). The controls were performed by replacing the primary antibody with either normal rabbit serum or PBS or by omitting any essential step of the reaction [28]. All of the ultrathin sections were observed and photographed using a JEM-1011 electron microscope (JEOL, JAPAN) equipped with a slow-scan digital camera at an accelerating voltage of 80 kV.

### Cell proliferation assay

The cell proliferation was measured using a BrdU cell proliferation enzyme-linked immunosorbent assay (ELISA) kit (Roche Diagnostics, Mannheim, DE). The BrdU assay was performed according to the manufacturer's protocol. The AGS cells were cultured in a 96-well multiplate at a density of 5 × 10^3^ cells per well in the serum-free medium described above for 24 h. The cells were then treated with the following: FBS (10% v/v), OB (100 nM and 200nM), OB-Ab (10 μg/mL), OB (100 nM) plus OB-Ab (10 μg/mL), CM24, CM48, CM24 plus OB-Ab (10 μg/mL) and CM48 plus OB-Ab (10 μg/mL) in fresh Ham's F12 (300 μL) for 48 h. BrdU incorporation was quantified using the spectrophotometric absorbance (370 nm) measured with a Reader VersaMaxPLUS. The mean absorbance of the control cells represented 100% cell proliferation, and the mean absorbance of the treated cells was related to the control values to determine sensitivity. In all cases, each experiment point was replicated eight times.

### Immunofluorescence detection of F-actin in AGS cells

The AGS cells were cultured at a density of 10 × 10^3^ cells per well in the culture medium described above on 8-well Nunc^®^ Lab-Tek^®^ II chamber slides covered with CC2 glass slide coverslips. After 2 days, the medium was renewed, and the cells were cultured in serum-free medium (300 μL) for 24 h. For GPR39-siRNA depleted cells, the AGS cells were transfected using Lipofectamine 2000 (Invitrogen; CA, US). Serum-starved cells were stimulated or not with obestatin (200 nM, 24 h) at 37°C. After 24 h, intact cells were fixed with 4% buffered paraformaldehyde-PBS for 30 min, washed, permeabilized with 0.25% Triton X-100 in PBS for 45 min, and blocked with PBST (1% Triton X-100, 1% Tween 20, 5% heat-inactivated normal goat serum, 1% BSA in PBS) for 30 min. For GPR39-siRNA depleted cells, the AGS cells were incubated with anti-GPR39 rabbit antibody diluted in 1% BSA in PBST (1 h, RT). After three washes with PBS, the cells were incubated with Phalloidin CruzFluor 594 and goat anti-rabbit IgG (H+L) secondary antibody Alexa Fluor^®^ 488 in 1% BSA in PBST (1 h, RT). DAPI was used to counterstain the cell nuclei (Invitrogen). Digital images of cells were acquired with a Zeiss Axio Vert.A1 fluorescence microscope (Carl Zeiss AG, Oberkochen, Germany).

### Cell-inverted invasion assay

The AGS cells were cultured as described above. The invasion assay was conducted as previously described [15]. Obestatin (200 nM) was used as a chemoattractant. The cells were allowed to migrate and invade into growth factor–reduced Matrigel for 24 h, pretreated with 50 μM cytarabine (to avoid proliferation), stained with 4 μmol/L calcein-acetoxymethyl ester (Invitrogen), and visualized by confocal microscopy (Leica TCS SP2 confocal microscope) using a × 10 objective. Optical sections were scanned at 5-μm intervals moving up from the underside of the membrane into the Matrigel. The fluorescence from each optical section was quantified with LCS Lite software (Leica Microsystems).

### Migration assay

The AGS cells were seeded on 6-well plates, grown to 100% confluence and then wounded with a sterile pipette tip to remove cells using linear scratches. The cells were washed and maintained in culture medium or culture medium plus 200 nM obestatin. The progress of migration was photographed immediately after injury and at 24 h after wounding, near the crossing point. The wound was calculated by tracing along the border of the scratch using ImageJ64 analysis software and the following equation: %wound closure = [[wound area (0 h) − wound area (x h)] / wound area (0 h)] × 100 [29].

### Immunoblot analysis of the EMT and the pro-angiogenic activation of obestatin in AGS cells

Serum-starved cells were stimulated with obestatin (200 nM) for 12, 24, and 48 h at 37°C. The medium was then aspirated, and the cells were lysed in ice-cold lysis buffer [RIPA buffer: 50 mM Tris-HCl pH 7.2, 150 mM NaCl, 1 mM EDTA, 1% (v/v) NP-40, 0.25% (w/v) Na-deoxycholate, protease inhibitor cocktail (1:100, Sigma Chemical Co., St. Louis, MO, US), phosphatase inhibitor cocktail (1:100, Sigma Chemical Co., St. Luis, MO, US)]. The soluble cell lysates were pre-cleared by centrifugation at 14,000 rpm for 15 min. The protein concentration was evaluated with the QuantiPro™ BCA Assay kit (Sigma Chemical Co., St. Louis, MO, US). The same amount of protein for each sample was separated on 10% sodium dodecyl sulfate (SDS)/polyacrylamide gels and transferred to nitrocellulose membranes (Bio-Rad, Hercules, CA, US). The blots were incubated with 5% non-fat milk in a Tris buffer solution containing Tween-20 (TBST) [20 mM Tris-HCl (pH 8.0), 150 mM NaCl, 0.1% (v/v) Tween-20, solution used for all incubation and washing steps] for 1 h. The blots were then incubated with the corresponding antibodies (E-cadherin, N-cadherin, β-catenin, vimentin, VEGF, VEGF-R2, PEDF and GAPDH) according to the manufacturer's instructions and were subsequently incubated with the corresponding peroxidase-conjugated IgG antibody. After washing, the signals were visualized using an ECL plus Western Blotting Detection System (Pierce ECL Western Blotting Substrate; Thermo Fisher Scientific, Pierce, Rockford, IL). The blots shown are representative of six experiments. Image processing was performed using the NIH Image Software ImageJ 1.49.

### siRNA silencing of gene expression and immunoblot analysis

The following double-stranded siRNA duplexes of GPR39 were used (Thermo Fisher Scientific, Dharmacon, Lafayette, CO, US; ON-TARGETplus SMART pool L-005569-00-0005, Human GPR39, NM_001508): 3′-UCCAAUAUGUCCAUCUGUA-5′, 3′-GCGCGAAACCAGCCAAUUC-5′, 3′-GAGGCUGAU UGUUGUGACA-5′, and 3′-AACCAGAUUCGGAGGAU CA-5′. A non-silencing RNA duplex was used as a control for all siRNA experiments. The AGS cells were transfected using Lipofectamine 2000 (Invitrogen; CA, US). Silencing was quantified by immunoblotting. Only experiments with verified silencing were used. Serum-starved cells were stimulated with obestatin (200 nM, 10 min for Akt and ERK1/2; and 200 nM, 24 h for Ki67) at 37°C. The immunoblot procedure is similar to that described above. The same amount of protein for each sample was separated on 10% (6% in the case of Ki67) SDS/polyacrylamide gels. The blots were incubated with the corresponding antibodies (p-Akt, p-ERK, Ki67, GPR39, β-actin). The blots shown are representative of three experiments. Image processing was performed using the NIH Image Software ImageJ 1.49.

### Human samples

The study protocol was approved by the local ethical committee (CAEI Galicia, 2009/118) and carried according to the Declaration of Helsinki. Histological classification of the gastric adenocarcinomas was performed according to the Lauren system [30]: 22 were intestinal type adenocarcinomas (with signet ring cells, *n* = 1), three diffuse type adenocarcinomas (with signet ring cells, *n* = 2), and three mixed (with signet ring cells, *n* = 2). Three levels of differentiation were used to classify the grading as follows: well in nine patients (WD), moderately in 10 patients (MD) and poorly differentiated in nine patients (PD). Pathological tumor staging was assessed according to the 7th edition of the American Joint Committee on Cancer TNM classification [31]. The pathological tissue samples were from gastric adenocarcinomas located in antrum and corpus. The surgical control specimens were located at least 3 cm from the adenocarcinoma. All of the examined control tissues originated from macro- and microscopically normal gastric mucosa.

### Immunohistochemistry detection of GPR39, obestatin and Ki67

Immunohistochemistry was performed according to the protocol previously described [32]. In brief, the samples (*n* = 28) were immersion-fixed in 10% buffered formalin for 24 h, dehydrated and embedded in paraffin using a standard procedure. The 5-μm-thick sections were mounted on xylanized microslides (Dako, Glostrup, DK), dewaxed and rehydrated. Antigen retrieval was carried out by heating in a microwave oven for 20 min at 750 W in 10 mM sodium citrate buffer (pH 6.0). For detection of obestatin and GPR39 the corresponding antibodies were used. Detection of Ki67 was performed using the same technique as described for immunocytochemistry. All of the sections were counterstained with HHS for 1 min. Pre-adsorption controls of the obestatin and GPR39 immunohistochemical technique were performed in gastric tissue. Negative controls (*n* = 3) were performed by substituting the antibody with PBS in one step of the technique or pre-adsorption of the antibody with the homologous antigen. For assessment of the GPR39 expression patterns, a scale from 0 to 3+ was used to express the incidence of positive immunoreaction in a semi-quantitative assay. Staining intensity was scored as 0 (absent), 1 (weak), 2 (moderate), or 3 (strong) by three independent observers. Ten different fields for each section were analyzed twice. For assessment of cell proliferation in the tumor tissue, the numbers of Ki67 were obtained by ACIS^®^ Assisted Quantitative Image Analysis. Photographs were taken using a Zeiss Observer Z1 microscope and Axiovision software (Carl Zeiss, Göttingen, GE). Two different observers performed the evaluation independently.

### Data analysis

All of the data are reported as the mean ± SE. Student's *t*-test was performed to assess the statistical significance of two-way analysis. For multiple comparisons, ANOVA was employed. Values of *P* < 0.05 were considered statistically significant (*). The strength of the relationship between the different parameters for GPR39 expression quantification was estimated by a Pearson correlation coefficient.
